# Resin-Induced Colonic *Pseudotumor*: Rare Complication from Chronic Use of Potassium Binders in a Hemodialysis Patient

**DOI:** 10.1155/2016/3692086

**Published:** 2016-02-29

**Authors:** Mary Bui, Shyan-Yih Chou, Pierre Faubert, Pablo Loarte, Ronny Cohen

**Affiliations:** ^1^Division of Nephrology and Hypertension, Brookdale University Hospital and Medical Center, Brooklyn, NY 11212, USA; ^2^Division of Hospital Medicine, Yale-New Haven Hospital, New Haven, CT 06510, USA; ^3^Department of Medicine/Cardiology Division, Woodhull Medical Center, Brooklyn, NY 11206, USA; ^4^Medicine Department, NYU School of Medicine, New York, NY 10016, USA

## Abstract

Potassium-binding resins are widely used in the treatment of hyperkalemia, mostly in the acute setting. Gastrointestinal adverse events, although reported, are not frequently seen due to its short course of use. This report describes a case involving an end-stage renal disease patient on hemodialysis who developed a colonic mass after being on sodium polystyrene sulfonate chronically for persistent hyperkalemia. Gastrointestinal symptoms developed late during the treatment rather than early as reported previously in the literature. This mass was mistaken for a carcinomatous lesion, which initiated an extensive work-up as well as hospitalization that nearly resulted in a subtotal colectomy.

## 1. Introduction 

Sodium polystyrene sulfonate is a cation-exchange resin that has been approved for the treatment of hyperkalemia in the United States since 1975. This medication is generally applied in the acute setting, but has gained popularity for use in patients with chronic hyperkalemia. Although generally well tolerated, use of sodium polystyrene sulfonate has been associated with a number of gastrointestinal complications that can be mistakenly confounded with pure gastrointestinal entities carrying a significant comorbidity.

## 2. Case Report 

**Figure 1:**
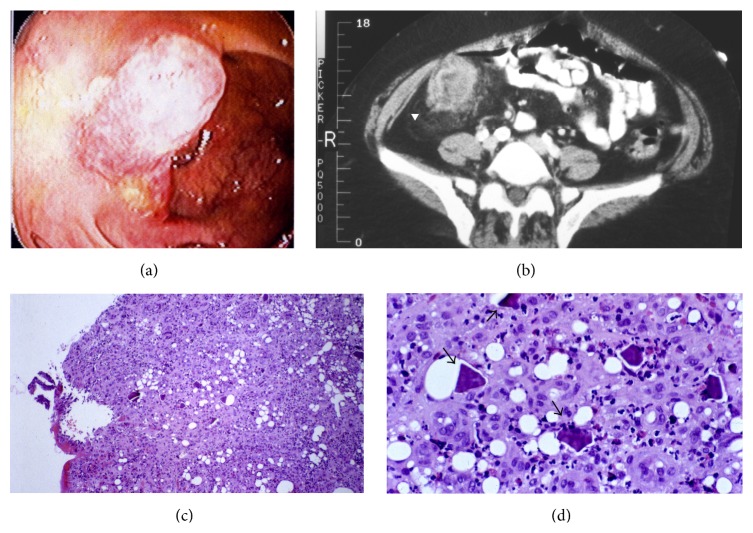
(a) A mass visualized in the cecum by colonoscopy. (b) Computed tomography of the abdomen (axial view) with a 5-cm mass identified in the right-lower abdominal area (white arrowhead). (c) Microscopic examination shows mucosal inflammation and presence of inclusion crystals (hematoxylin-eosin stain) (×100). (d) Sodium polystyrene sulfonate crystals (black arrows) surrounded by inflammatory cells (hematoxylin-eosin stain) (×400).

A 70-year-old woman with end-stage renal disease secondary to hypertensive nephrosclerosis had been on hemodialysis for 6 years. Her past medical history included left mastectomy, diverticulosis, parathyroidectomy due to secondary hyperparathyroidism, and coronary artery disease for which she received a 4-vessel coronary artery bypass graft. Her dialysis was scheduled 3 times per week via an upper extremity arteriovenous graft, each session of 3 hours and 45 minutes and with a dry weight goal of 68.6 kg. She was in her usual state of health until approximately one year earlier when the results of routine laboratory tests revealed persistently elevated potassium levels (Table 1). In addition to the use of a low-potassium dialysate, intense efforts were made to modify her diet, but failed to correct hyperkalemia. Her medication list did not include any ACE inhibitor or other medications that could potentially cause hyperkalemia (Table 2). She was initiated on sodium polystyrene sulfonate (in water suspension) at a dose of 15 grams twice daily after meals and continued taking it for a year. The patient never received sodium polystyrene sulfonate in sorbitol as an oral preparation or as a retention enema. One week prior to her hospitalization, she began to have occasional constipation with intermittent loose bowel movements and the stool guaiac tested positive. The patient underwent an esophagogastroduodenoscopy and colonoscopy. She was found to have gastritis and duodenitis in the upper gastrointestinal tract, with a mass on the upper lip of the ileocecal valve (Figure 1(a)) and internal hemorrhoids. A biopsy of the mass was done. An abdominal computed tomography (Figure 1(b)) identified the presence of an ill-defined 5-cm cecal mass with localized inflammatory changes. She was admitted to the hospital for a subtotal colectomy. On the morning of the scheduled surgery, the histological examination did not reveal any evidence of carcinoma and the surgical intervention was postponed. She underwent a second colonoscopy with biopsy, which again did not show tumor cells. However, the histological examination revealed the presence of rhomboid mosaic crystals, surrounded by a mixed population of inflammatory cells including eosinophils, macrophages, plasma cells, and lymphocytes (Figures 1(c) and 1(d)). There was also presence of fibroblasts, collagenous materials, and mucosal erosion. It stained negative for the presence of calcium, implying that the mass was not the result of the high calcium intake. The surgery was cancelled and the patient was discharged from the hospital.

## 3. Discussion 

Sodium polystyrene sulfonate is a cation-exchange resin, mostly used in an acute episode of hyperkalemia. It has also been utilized in the setting of chronic hyperkalemia that results from the necessary use of various medications or from dietary indiscretion in patients with chronic kidney disease. The resin can be given orally or as a retention enema. When administered orally, the resin releases sodium cations in exchange for potassium ions and is eventually eliminated in the feces. The end result is that serum potassium is lowered over a period of hours to days [1, 2].

Although generally well tolerated, polystyrene sulfonate in a water suspension may cause a number of gastrointestinal complications, including constipation, bowel bezoars comprising of sodium polystyrene sulfonate crystals and gut debris, fecal impaction, and bowel obstruction [2, 3]. To improve drug tolerance, sorbitol has been used as a vehicle for the administration of sodium polystyrene sulfonate to promote an osmotic diarrhea. The addition of sorbitol to the suspension, however, could potentially result in extensive mucosal injury with pathological findings of transmural necrosis [2, 4–7].

Sodium polystyrene sulfonate has also been reported to mimic a variety of entities in the upper gastrointestinal tract including esophageal candida and esophageal carcinoma [2]. In a review of 58 cases of gastrointestinal adverse events associated with the use of sodium polystyrene sulfonate (oral and rectal route, 80 and 20%, resp.), the colon was the most common site of injury with a 78% of cases found there [8]. The cecum was the less involved area (10%) and the presence of sodium polystyrene sulfonate crystals within the injured areas represented a “footprint” of its use. Crystals were found in 90% of the cases described, and the most common symptomatology was abdominal pain, and less frequently diarrhea and gastrointestinal bleeding. The majority of these 58 cases involved the use of sodium polystyrene sulfonate with sorbitol (41 cases) and all the adverse events were related to its use in the acute setting. The study was limited by incomplete information in some of the reported cases and the risk attributable to its use was unable to be estimated [8]. The existing literature does describe only one case where the patient developed a gastrointestinal complication after a prolonged use of sodium polystyrene sulfonate. The importance of identifying the sodium polystyrene sulfonate crystals in these situations is evident in order to avoid unnecessary investigations, and possibly deleterious interventions [9]. Here, we report a mistaken diagnosis that could have resulted in unnecessary subtotal colectomy for a cecal mass.

Most of the cases reported to date are those where sorbitol is used. The exact mechanisms of injuries in these cases are unknown; it has been suggested that certain factors may predispose patients to acute injury. These factors include uremia, changes in blood volume associated with dialysis or surgery, low gut motility with consequent prolonged exposure to the toxic effect of the resin in sorbitol, and posttransplant immunosuppressive therapy [2–10]. Mucosal injury was noted in those patients who received retention enemas as well as those who took the drug orally. The injury can involve upper or lower gastrointestinal tract. The lesions range from erosive ulcers of the esophagus and stomach, as well as colonic injuries such as bleeding or perforated cecal ulcers, necrotizing ulcerative enterocolitis, ischemic colitis, and diverticular perforation [2, 5–10].

Lillemoe and colleagues [11] suggested that the sorbitol used in enemas, and not the sodium polystyrene sulfonate itself, might cause the gut necrosis. This hypothesis was tested in normal and uremic rats that were subject to enemas containing saline, sodium polystyrene sulfonate in water, sodium polystyrene sulfonate in sorbitol, and sorbitol alone. In both normal and uremic rats, only the rats that received enemas of sorbitol alone or sorbitol-containing resin developed extensive transmural necrosis, whereas the rats exposed to sodium polystyrene sulfonate alone developed, at worst, mucosal erythema. The mechanism of injury may be related to a direct toxic effect of an increase in local prostaglandin activity induced by sorbitol rather that sodium polystyrene sulfonate [12].

In our case, the lack of sorbitol in the resin formulation might have reduced the likelihood of bowel necrosis and other types of lesions; however, it could have contributed to a slow gastrointestinal transit. Sorbitol is a laxative; therefore, a slow transit time would increase the interaction between the resin and the colonic mucosa. Consequently, mucosal erosion develops, possibly caused by the crystals in the gastrointestinal lumen before “internalization” occurs. The crystals end up internalizing within the mucosa or embedded within a pseudotumor composed of dense inflammatory cells, giving the aspect of a luminal mass. Consistent with this possibility, a case report described polystyrene sulfonate embedded in a perihepatic inflammatory pseudotumor adjacent to a colostomy site [9]. It has also been demonstrated that simple inoculation of any tissue with sodium polystyrene sulfonate leads to an acute inflammatory reaction within 24 hours [8]. Finally, it is possible that the concomitant oral use of calcium carbonate could be an additional factor for the lesion to occur. Sodium polystyrene sulfonate, when administered in a simple solution, tends to bind calcium ions and produces fecal impaction and bowel obstruction [13].

At the end, the question remains regarding whether other therapeutic options would have prevented this from happening. In the market, there is another available resin, calcium polystyrene sulfonate, but it is also associated with similar adverse gastrointestinal events, including colonic necrosis and perforation when used with or without sorbitol [14–16]. Recently, two new potassium binders, patiromer and sodium zirconium cyclosilicate, have been reported to effectively treat hyperkalemia in patients with chronic kidney disease not yet on dialysis [17, 18]. These drugs exchange dietary potassium for sodium or calcium in the gut to reduce potassium absorption. Limitations of these studies include a relatively short treatment period and lack of comparison with the existing potassium binders. Patients with severe hyperkalemia were also excluded from these clinical trials. The long-term efficacy and safety of these novel potassium binders are yet to be determined in dialysis patients.

## 4. Conclusion

Serious injuries to the gastrointestinal tract caused by sodium polystyrene sulfonate are rare and varied, mostly in the form of mucosal erosion, gut necrosis, stenosis, or perforation and can occur early or late after its administration. Our case is unique in that a colonic mass developed late after chronic use of sodium polystyrene sulfonate. It was mistaken for a carcinomatous lesion that nearly resulted in unnecessary colectomy. This highly uncommon occurrence should remind clinicians of the possibility of a pseudotumor. The presence of crystals in the biopsy may serve as a “hallmark” of resin-induced injuries.

## Figures and Tables

**Table 1:** Values of predialysis laboratory blood tests.

Parameter	1 Year before admission	1 Month before admission
Potassium	6.5 mmol/L	5.4 mmol/L
Bicarbonate	22.6 mmol/L	23 mmol/L
Creatinine	6.7 mg/dL	5.8 mg/dL
BUN	42 mg/dL	39 mg/dL
Calcium	8.9 mg/dL	9 mg/dL
Phosphorus	2.1 mg/dL	2.3 mg/dL
Albumin	3.4 g/dL	3.3 g/dL
Hemoglobin	9.7 g/dL	9.9 g/dL
Iron	48 mcg/dL	59 mcg/dL
TIBC	235 mcg/dL	185 mcg/dL
Iron saturation	15%	21%
Ferritin	326 ng/mL	659 ng/mL
sp*Kt/V*	1.90	1.93

sp*Kt/V*: single-pool *Kt/V*.

**Table 2:** Medications received by the patient.

Medication	Dose and frequency
Ca^2+^ carbonate	1,500 mg three times daily
Aspirin	81 mg daily
Clopidogrel	75 mg daily
Metoprolol	50 mg daily
Amlodipine	5 mg daily
Ergocalciferol	50,000 mcg weekly
Darbepoetin alfa^*∗*^	60 mcg IV weekly
Sodium ferric gluconate^*∗*^	125 mg IV weekly
Sodium polystyrene sulfonate	15 g twice daily
Dialysate 2K^*∗*^	3 times weekly

^*∗*^Administered during hemodialysis sessions; IV: intravenous administration; dialysate 2K: dialysate potassium concentration, 2 mmol/L.
